# Tonantzitlolone cytotoxicity toward renal cancer cells is PKCθ- and HSF1-dependent

**DOI:** 10.18632/oncotarget.4676

**Published:** 2015-07-20

**Authors:** Carole Sourbier, Bradley T. Scroggins, Philip Z. Mannes, Pei-Jyun Liao, Karsten Siems, Dietmar Wolf, John A. Beutler, W. Marston Linehan, Leonard Neckers

**Affiliations:** ^1^ Urologic Oncology Branch, Center for Cancer Research, National Cancer Institute, Bethesda, MD 20892, USA; ^2^ Radiation Oncology Branch, Center for Cancer Research, National Cancer Institute, Bethesda, MD 20892, USA; ^3^ AnalytiCon Discovery GmbH, D-14473 Potsdam, Germany; ^4^ Molecular Targets Laboratory, Center for Cancer Research, National Cancer Institute, Frederick, MD 21702, USA

**Keywords:** englerin A, bryostatin, renal tumors, RCC, PKCθ

## Abstract

Elucidating the targets and mechanism of action of natural products is strategically important prior to drug development and assessment of potential clinical applications. In this report, we elucidated the main targets and mechanism of action of the natural product tonantzitlolone (TZL) in clear cell renal cell carcinoma (CCRCC). We identified TZL as a dual PKCα and PKCθ activator *in vitro*, although in CCRCC cells its activity was mostly PKCθ-dependent. Through activation of PKCθ, TZL induced an insulin resistant phenotype by inhibiting IRS1 and the PI3K/Akt pathway. Simultaneously, TZL activated the heat shock factor 1 (HSF1) transcription factor driving glucose dependency. Thus, similar to the selective PKCθ activator englerin A, TZL induces a metabolic catastrophe in CCRCC, starving cells of glucose while simultaneously increasing their glycolytic dependency.

## INTRODUCTION

Multiple signaling pathways are involved in regulating cell metabolism. Several, including components of the insulin pathway, are regulated by members of the protein kinase C (PKC) family. The multiple isoforms of PKC directly or indirectly regulate diverse aspects of metabolism in an isoform specific manner [1, 2]. For example, PKCθ and -ε induce insulin resistance by inhibiting insulin receptor substrate 1 (IRS1) and the insulin pathway, while PKCδ and -λ have the opposite effect and stimulate the insulin pathway by activating IRS1 [2]. PKC isoforms can also regulate additional aspects of cell metabolism, e.g. PKCζ has recently been shown to regulate glutamine metabolism [3]. Therefore, developing isoform-specific regulators of PKCs might have useful clinical applications for metabolically deregulated diseases, including most epithelial cancers. One example of this is the recent identification of the natural product englerin A (EA; C_26_H_34_O_6_) as a potent and selective PKCθ activator [4]. This finding is furthering our understanding of the role played by PKCθ in tumor tissues and identifies PKCθ interaction with the heat shock factor 1 (HSF1) transcription factor as a critical link between cell metabolism and cell stress response.

Tonantzitlolone (C_26_H_40_O_7_) is a diterpene ester identified in 1990 from the native Mexican plant *Stillingia sanguinolenta* Müll. Arg. (Euphorbiaceae). The initial interest towards investigating *S. sanguinolutea* biomedical properties comes from native Mexican, Navajo, and Creek traditional use as a medicinal plant [5]. Reports have since addressed TZL's structure, absolute configuration, and total synthesis [6, 5]. Although preliminary data have suggested that TZL might have an anticancer effect, a thorough characterization of the biological effects of TZL is lacking and its targets as well as its mechanisms of action are yet to be discovered. In this report, our goal was to identify TZL primary targets and to characterize the molecular effect of TZL on tumor cells.

## RESULTS

### Tonantzitlolone is a PKC activator with weak isoform selectivity

**Figure 1:**
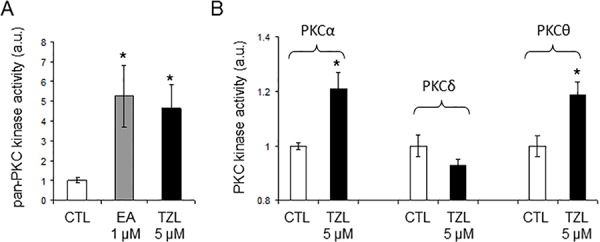
Tonantzitlolone is a PKC activator **A.** Pan-PKC kinase activity was assessed by pan-PKC kinase assay using 30 μg of whole cell extracts from the CCRCC cell line 786-0. Lysates were treated with either DMSO or TZL (5 μM; 1 hr at 30°C). **B.** Assessement of PKC isoform selectivity was performed following a similar protocol but using purified proteins for PKCα, -δ, and -θ (5 ng). *p < 0.05; EA: englerin A; TZL: Tonantzitlolone.

Analysis of NCI 60 selectivity data indicated that TZL had a pattern of response very similar to englerin A, with a Pearson correlation of 0.91 at the GI-50 level of response; Table 1 [8]. All other pure compound correlations (n = 9) above 0.8 were to englerin analogues, with one exception (#741581, sodwanone W [7]). In addition, quantitative structure activity relationship (QSAR) analysis revealed that TZL is structurally similar to the protein kinase C (PKC) activator bryostatin 1 and analogues, suggesting that TZL is likely to have an effect on the protein kinase C (PKC) family (Tables 2 and 3; [8]). To validate these results, we used a pan-PKC kinase assay. Whole protein extracts of the clear cell renal cell carcinoma (CCRCC) cell line 786-0 were directly treated with TZL (5 μM; 1 h at 30°C). We used the anticancer agent EA, a selective PKCθ activator [4], as a positive control. This assay revealed that TZL is a potent PKC activator (Figure 1A). Because of the variable functions of the different PKC isoforms, we assessed TZL isoform selectivity by *in vitro* kinase assay using purified proteins. As shown in Figure 1B, TZL efficiently activated PKCθ and PKCα, but not PKCδ.

**Table 1:** Compounds correlated to tonantzitlolone at GI-50 level in NCI 60 screen

NSC#	Pearson Correlation	Compound type
746861	0.91	Englerin A
767580	0.88	EA analog
780423	0.87	EA analog
741581	0.86	sodwanone W
782488	0.85	EA analog
782490	0.84	EA analog
778312	0.84	EA analog
782489	0.83	EA analog
767579	0.83	EA analog
782483	0.83	EA analog

**Table 2:** List of compounds structurally similar to tonantzitlolone (based on QSAR modeling)

#	Compound in database	Structure	Similarity, %
1	Bryostatin Analogue (8)	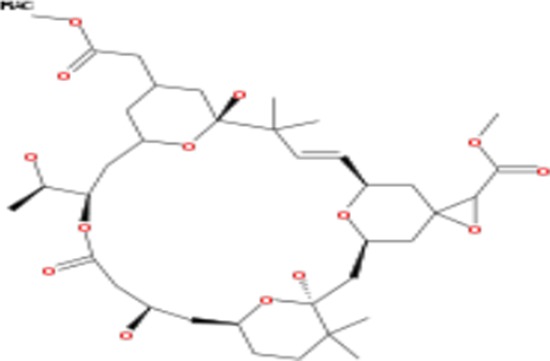	82.11
2	Bryostatin Analogue (9)	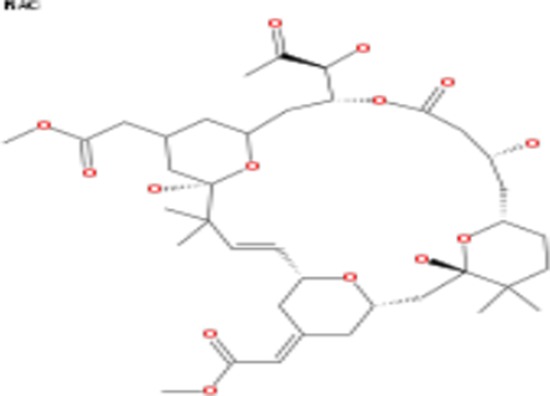	80.41
3	Bryostatin Analogue (1)	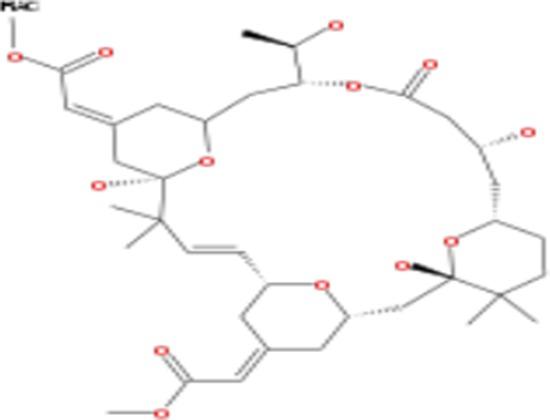	78.35
4	Bryostatin Analogue (6)	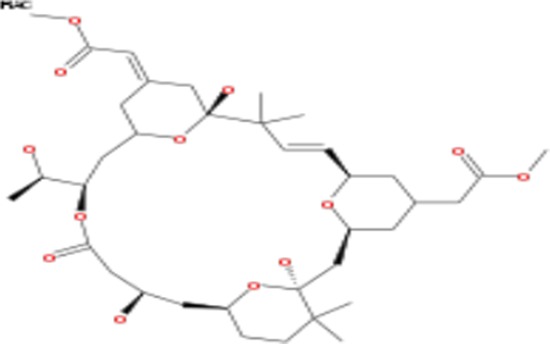	78.12
5	Bryostatin Analogue (3)	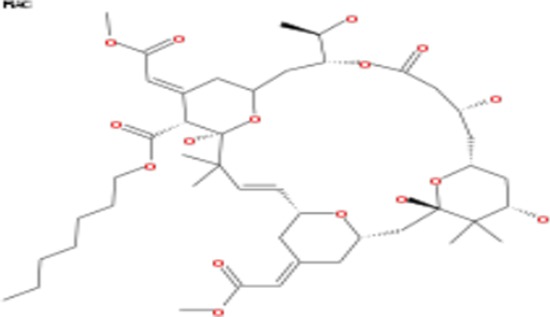	78
6	Ginkgolide (22)	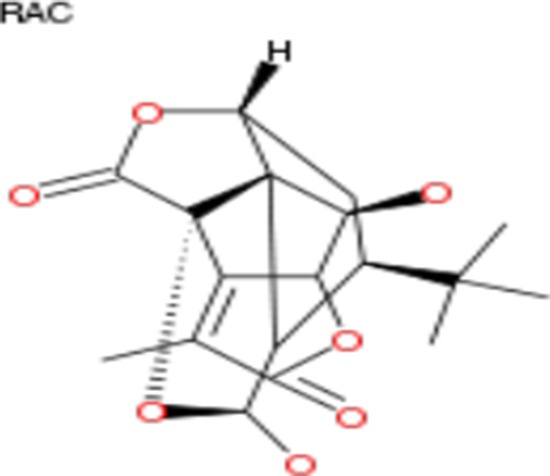	77.78
7	2-[3-(1, 3-dioxan-2-yl)-1, 1-dimethyl-(E)-2-propenyl]-2-hydroxy-6-[3-hydroxy-2-methylcarbonyloxy-(2R, 3R)-butyl]-4-methyloxycarbonylmethyl-(2S, 3S, 6S)-tetrahydro-2H-3-pyranyl octanoate	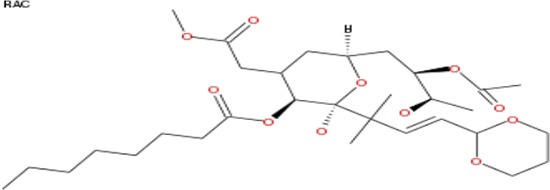	77.32
8	Bryostatin Analogue (2)	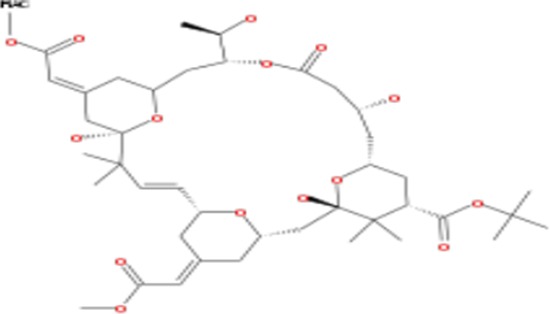	76.77

**Table 3:** List of tonantzitlolone predicted targets (QSAR; based on [8])

Predicted Target	Compound of reference	Similarity (%)
PKC-lambda/iota	Bryostatin Analogue (8)	82.11
	Bryostatin Analogue (9)	80.41
	Bryostatin Analogue (1)	78.35
	Bryostatin Analogue (6)	78.12
	Bryostatin Analogue (3)	78
	2-[3-(1, 3-dioxan-2-yl)-1, 1-dimethyl-(E)-2-propenyl]-2-hydroxy-6- [3-hydroxy-2-methylcarbonyloxy-(2R, 3R)-butyl]-4-methyloxycarbonylmethyl-(2S, 3S, 6S)-tetrahydro-2H-3-pyranyl octanoate	77.32
PKC-epsilon	Bryostatin Analogue (8)	82.11
	Bryostatin Analogue (9)	80.41
	Bryostatin Analogue (1)	78.35
	Bryostatin Analogue (6)	78.12
	Bryostatin Analogue (3)	78
	2-[3-(1, 3-dioxan-2-yl)-1, 1-dimethyl-(E)-2-propenyl]-2-hydroxy-6-[3-hydroxy-2-methylcarbonyloxy-(2R, 3R)-butyl]-4-methyloxycarbonylmethyl-(2S, 3S, 6S)-tetrahydro-2H-3-pyranyl octanoate	77.32
PKC-zeta	Bryostatin Analogue (8)	82.11
	Bryostatin Analogue (9)	80.41
	Bryostatin Analogue (1)	78.35
	Bryostatin Analogue (6)	78.12
	Bryostatin Analogue (3)	78
	2-[3-(1, 3-dioxan-2-yl)-1, 1-dimethyl-(E)-2-propenyl]-2-hydroxy-6-[3-hydroxy-2-methylcarbonyloxy-(2R, 3R)-butyl]-4-methyloxycarbonylmethyl-(2S, 3S, 6S)-tetrahydro-2H-3-pyranyl octanoate	77.32
PKC-delta	Bryostatin Analogue (8)	82.11
	Bryostatin Analogue (9)	80.41
	Bryostatin Analogue (1)	78.35
	Bryostatin Analogue (6)	78.12
	Bryostatin Analogue (3)	78
	2-[3-(1, 3-dioxan-2-yl)-1, 1-dimethyl-(E)-2-propenyl]-2-hydroxy-6-[3-hydroxy-2-methylcarbonyloxy-(2R, 3R)-butyl]-4-methyloxycarbonylmethyl-(2S, 3S, 6S)-tetrahydro-2H-3-pyranyl octanoate	77.32
PKC-alpha	Bryostatin Analogue (8)	82.11
	Bryostatin Analogue (9)	80.41
	Bryostatin Analogue (1)	78.35
	Bryostatin Analogue (6)	78.12
	Bryostatin Analogue (3)	78
	2-[3-(1, 3-dioxan-2-yl)-1, 1-dimethyl-(E)-2-propenyl]-2-hydroxy-6-[3-hydroxy-2-methylcarbonyloxy-(2R, 3R)-butyl]-4-methyloxycarbonylmethyl-(2S, 3S, 6S)-tetrahydro-2H-3-pyranyl octanoate	77.32
PKC-alpha	Bryostatin Analogue (2)	76.77
PKC-eta	Bryostatin Analogue (8)	82.11
	Bryostatin Analogue (9)	80.41
	Bryostatin Analogue (1)	78.35
	Bryostatin Analogue (6)	78.12
	Bryostatin Analogue (3)	78
	2-[3-(1, 3-dioxan-2-yl)-1, 1-dimethyl-(E)-2-propenyl]-2-hydroxy-6-[3-hydroxy-2-methylcarbonyloxy-(2R, 3R)-butyl]-4-methyloxycarbonylmethyl-(2S, 3S, 6S)-tetrahydro-2H-3-pyranyl octanoate	77.32
PKC-gamma	Bryostatin Analogue (8)	82.11
	Bryostatin Analogue (9)	80.41
	Bryostatin Analogue (1)	78.35
	Bryostatin Analogue (6)	78.12
	Bryostatin Analogue (3)	78
	2-[3-(1, 3-dioxan-2-yl)-1, 1-dimethyl-(E)-2-propenyl]-2-hydroxy-6 -[3-hydroxy-2-methylcarbonyloxy-(2R, 3R)-butyl]-4-methyloxycarbonylmethyl-(2S, 3S, 6S)-tetrahydro-2H-3-pyranyl octanoate	77.32
PKC-theta	Bryostatin Analogue (8)	82.11
	Bryostatin Analogue (9)	80.41
	Bryostatin Analogue (1)	78.35
	Bryostatin Analogue (6)	78.12
	Bryostatin Analogue (3)	78
	2-[3-(1, 3-dioxan-2-yl)-1, 1-dimethyl-(E)-2-propenyl]-2-hydroxy-6- [3-hydroxy-2-methylcarbonyloxy-(2R, 3R)-butyl]-4-methyloxycarbonylmethyl-(2S, 3S, 6S)-tetrahydro-2H-3-pyranyl octanoate	77.32

### Tonantzitlolone is a potent anti-tumor agent

**Figure 2:**
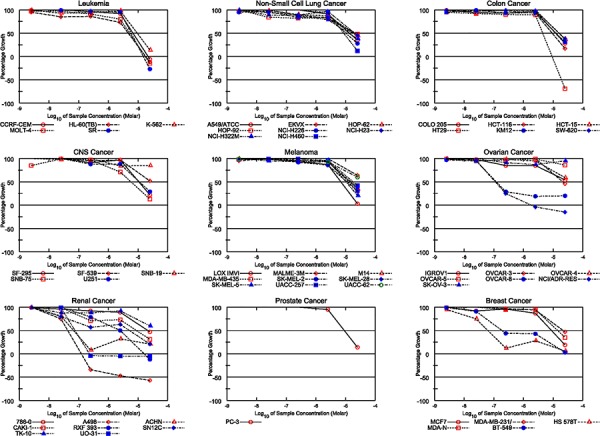
Tonantzitlolone has an anti-tumor effect, especially in kidney cancer cells Similarly to englerin A [8], the cytotoxic effect of TZL in the NCI-60 tumor cell panel shows a preference for renal cancer cell lines.

**Figure 3:**
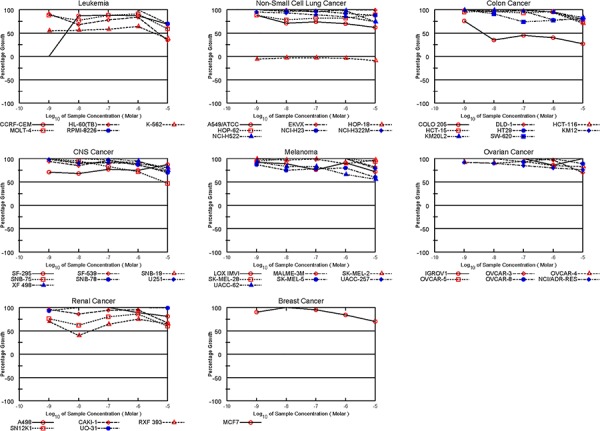
Effect of Bryostatin in the NCI60 cell line screen Data about the cytotoxic effect of the pan-PKC activator bryostatin in the NCI60 tumor cell lines panel was extracted from the NCI60 database. No renal selectivity was observed.

To further assess the biologic effect of TZL on the viability of tumor cells, we used the NCI-60 screen, a platform containing 60 different cancer cell lines [9]. Interestingly, TZL displayed a preferred cytotoxicity towards renal cancer cells (Figure 2). In a similar screen, EA also displayed renal selectivity [10]; however this was not seen with the pan-PKC activator bryostatin 1 (Figure 3; bryostatin's data were extracted from the public repository database CellMiner; NSC#339555) [11]. These data suggest that the difference in toxicity might be due to a variation in isoform selectivity, and that TZL's effect might be PKCθ-dependent.

### Tonantzitlolone cytotoxicity in renal cancer cells is PKCθ-dependent

**Figure 4:**
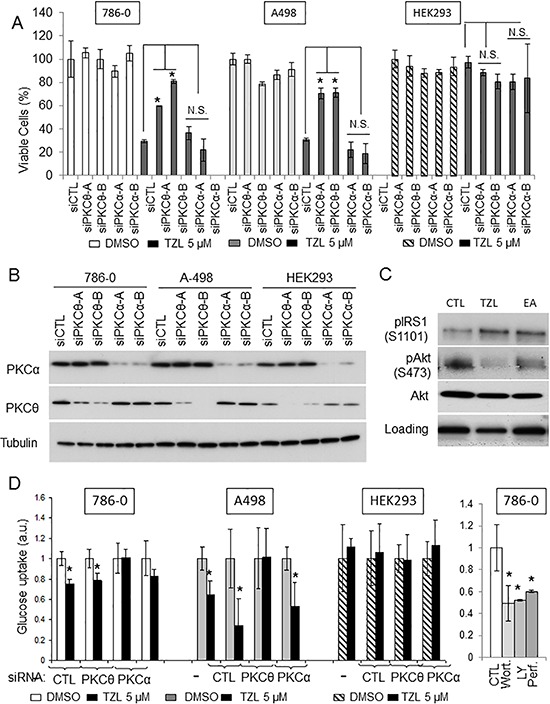
Tonantzitlolone effect is PKCθ-dependent **A.** Viability of VHL-deficient cell lines A498 and 786-0 was assessed following transient silencing of PKCθ or PKCα using 2 distinct small interference RNAs. The embryonic kidney cell line HEK293 was used as control. **B.** Silencing efficiency was assessed by immunoblotting. **C.** Effect of TZL on the insulin pathway was assessed by immnublotting. EA was used as a positive control. **D.** Effect of TZL on glucose uptake was assessed in VHL-deficient 786-0 and A498 cells. The embryonic kidney cell line HEK293 and treatment of 786-0 cells with the PI3K/Akt inhibitors wortmannin, LY294002 and perifosine (5 μM; 4 h) were used as controls. *p < 0.05; N.S.: non-significant; EA: englerin A; TZL: tonantzitlolone; Wort: wortmannin; LY: LY294002; Perf: perifosine.

We then sought to assess whether TZL cytotoxicity was dependent on a particular PKC isoform. Because of the direct effect of TZL on PKCθ and PKCα purified proteins (Figure 1B), we separately silenced these two kinases in 786-0 and A498 cells using small interference RNA prior to exposure to TZL. As shown in Figure 4A-B, silencing of PKCθ partially rescued the cells from TZL cytotoxicity while silencing of PKCα did not. This suggests that PKCθ expression is necessary to mediate TZL cytotoxicity. HEK293 cells are not sensitive to TZL (Figure 4A) and express low levels of PKCθ compared to 786-0 and A498, but express robust levels of PKCα (Figure 4B; [6]), suggesting that TZL's effect in CCRCC cells is likely mediated by PKCθ. To further evaluate the effect of TZL on PKCθ signaling pathway in CCRCC cells, we treated 786-0 and A498 CCRCC cell lines with TZL and assessed the effect of treatment on the insulin pathway. As shown in Figure 4C, TZL increased phosphorylation of IRS1 on serine 1101 (a PKCθ-dependent phosphorylation site that inhibits IRS1 activity) and decreased Akt phosphorylation. Moreover, glucose uptake was also inhibited after TZL treatment in a PKCθ-dependent manner (5 μM, 16 h; Figure 4D), suggesting that TZL limits tumor cells access to glucose by inducing an insulin resistant phenotype *via* activation of PKCθ.

**Figure 5:**
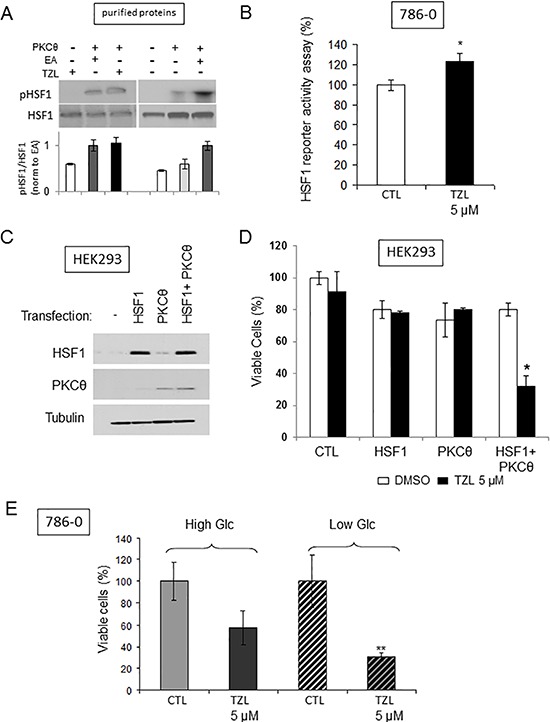
Tonantzitlolone activates HSF1 and induces glucose dependency **A.** Radioactive kinase activity assay was performed to assess the effect of TOZL (5 μM) on PKCθ-mediated phosphorylation of HSF1 in the presence of 6 μCi (0.2 μM) of [^32^P]-ATP and 10 μM non-radioactive ATP using purified PKCθ and purified HSF1 (50 ng of purified proteins; treatment for 30 min at 30°C). EA (0.1 μM) was used as positive control [4]. **B.** HSF1 transcriptional activity following TZL treatment (5 μM, 24 h) was assessed using a luciferase reporter assay in 786-0 cells. **C.** Overexpression efficiency of HSF1 and PKCθ in HEK293 cells was assessed by immunoblotting 24 h post-transfection. **D.** Effect of TZL (5 μM) on viability 24 h after treatment of HEK293 transfected with HSF1 and/or PKCθ (cells treated 24 h post-transfection). **E.** 786-0 cell viability was assessed following TZL treatment (5 μM) in high glucose (4.5 g/L) or low glucose (1 g/L) media. *p < 0.05; EA: englerin A; TZL: Tonantzitlolone.

We previously established a link between PKCθ and HSF1, a transcription factor known to induce tumor glucose dependency [12, 13], namely direct phosphorylation and heat-shock-independent activation of HSF1 by PKCθ [4]. We thus investigated whether TZL also induced a direct heat-shock-independent activation of HSF1. Using an *in vitro* kinase assay with purified PKCθ and HSF1 proteins, we demonstrated that TZL was able to induce PKCθ-dependent HSF1 phosphorylation (Figure 5A). Furthermore treatment of 786-0 cells with TZL (5 μM, 24 h) increased HSF1 transcriptional activity as measured with a luminescence-reporter plasmid assay (Figure 5B) and similarly to effects observed with EA, TZL cytotoxicity was observed only when HSF1 and PKCθ were simultaneously overexpressed in HEK293 cells (Figure 5C-5D). Also, TZL's cytotoxicity was dependent on the extracellular glucose concentration (Figure 5E). Indeed, in low glucose media (1 g/L), TZL was significantly more cytotoxic than in high glucose media (4.5 g/L). Together these data demonstrate the ability of TZL to induce glucose dependency in CCRCC cell lines.

## DISCUSSION

Understanding the intertwined role that members of the PKC family play in signaling pathways and cancer has the potential to lay the foundation for development of novel targeted therapies. The resurgent interest of the scientific community in understanding the molecular mechanisms regulating the metabolism of cancer cells has shed a new light on the role of PKC in regulating metabolic processes, especially in tumor cells. Here we report that the natural product TZL is a potent PKC activator with tumor cytotoxicity that is mostly mediated by the novel PKC isoform PKCθ. By activating PKCθ, TZL inhibits the insulin pathway while activating HSF1 and glucose dependency.

Clear cell renal cell carcinoma is the most common type of both sporadic and hereditary kidney cancer. It is primarily induced by mutations occurring in the tumor suppressor gene *VHL* [14]. Defective VHL protein expression leads to stabilization of hypoxia-inducible factors 1and 2 [15, 16], increased DNA damage [17], and to activation of the PI3K/Akt pathway stimulating cell proliferation and tumor growth [18]. Thus VHL-deficient cells display a pseudo-hypoxic phenotype with a restructured metabolism oriented toward aerobic glycolysis and favoring anabolic growth [12]. Interestingly, PKC members have been shown to be induced by hypoxia and novel PKC isoforms are known to interact with VHL, suggesting that VHL might directly regulate the expression of some of the PKC isoforms. Also, besides a well-characterized role in regulating cell cycle and survival [19, 20, 21], PKC isoforms are also critical in regulating metabolic processes [2, 3]. Hence, CCRCC cells are useful tools for studying PKC isoform functions.

Although structurally different from the natural product EA [5, 10], TZL has a similar profile in the NCI-60 screen suggesting that the two compounds might share a similar mechanism of action. Using *in vitro* kinase assays, we showed that TZL activated PKCθ and not PKCδ and that its cytotoxicity was PKCθ dependent in CCRCC cells. Because of the limited expression profile of PKCθ to some solid tumors and immune cells, and the fact that HSF1 is often overexpressed in tumors, TZL might only be lethal to PKCθ-expressing, HSF1- addicted glycolytic tumor cells.

Despite the well-characterized role of HSF1 in cancer development and progression [13, 12, 22, 23], in the context of PKCθ activation and the inhibition of the insulin pathway, non-heat-shock HSF1 activation appears to provide a therapeutic advantage. HSF1 is a tumor growth facilitator that enhances tumor glucose dependence via a heat-shock-independent transcriptional program [12]. Since TZL activates HSF1 and its cytotoxicity is inversely related to extracellular glucose concentration (Figure 5E), the fact that HSF1 increases glucose dependency might provide a rationale for why activation of HSF1 underlies the cytotoxicity of PKCθ activators such as TZL.

In conclusion, our data suggest that TZL and the PKCθ activator EA share a similar mechanism of action in CCRCC cell lines—that is PKCθ activation that leads to a metabolic catastrophe by simultaneously inhibiting the insulin pathway *via* inhibitory phosphorylation of IRS1 while inducing glucose addiction *via* HSF1 activation. These data also further support PKCθ as a strategic therapeutic target for patients with highly glycolytic tumors, such as CCRCC.

## MATERIALS AND METHODS

### Reagents

Tonantzitlolone was generously supplied by AnalytiCon Discovery GmbH (Postdam, Germany). Englerin A was isolated as described from *Phyllanthus engleri*. [9]. Complete mini-protease inhibitor cocktail tablets were purchased from Roche (Indianapolis, IA, USA). Purified HSF1, PKC-θ, -α, and -δ were purchased from EnzoLife Sciences (Farmingdale, NY, USA). PI3K and Akt inhibitors wortmannin, LY294002 and perifosine were from Selleck Chemicals (Houston, TX, USA).

### Prediction of tonantzitlolone targets

Prediction of TZL target was performed using the software Metadrug (Genego Inc, Carlsbad, CA, USA), a systems pharmacology platform using QSAR modeling to analyze and compare biological effects of small molecule, as previously described [4]. The COMPARE algorithm was essentially as described in [8].

### Non-radioactive PKC kinase assay

PKC kinase activity of cell lysates was measured using the pan-PKC activity assay from EnzoLife Sciences. Cells were lysed in TNESV lysis buffer (50 mM Tris, 1% Nonidet P-40, 2 mM EDTA, 100 mM NaCl and 2 mM Na_3_VO_4_). After centrifugation (15 min; 15,000 rpm; 4°C), clarified supernatant was incubated with 10 μM TZL in the kinase buffer provided by the manufacturer (1 h at 30°C with 10 μM ATP). The reaction was stopped and the phosphorylation of a PKC substrate was measured by spectrophotometry as indicated in the manufacturer protocol. PKCα, -δ, and θ kinase assays were performed in a similar manner using 5 ng of purified PKC proteins (incubation for 1 h at 30°C with 10 μM ATP).

### Radioactive *in vitro* kinase assay

Purified HSF1 (50 ng) was incubated with purified PKCθ (50 ng) in presence or absence of TZL (1 μM). Reactions were initiated by the addition of 10 μM nonradioactive ATP and 6 μCi (0.2 μM) of [^32^P]-ATP and incubated at 30°C for 30 min with periodic mixing. Proteins in the kinase reactions were separated by SDS-PAGE and transferred to PVDF membrane. Phosphorylation of HSF1 was assessed by radiography of PVDF membranes. Total HSF1 was then immunoblotted to ensure equal loading.

### Cell lines and cell culture

The sporadic VHL-deficient kidney tumor cell lines 786-0 and A498, and the embryonic kidney epithelial cell line HEK293 were all purchased from American Type Culture Collection (ATCC; Manassas, VA, USA). Cells were cultured in Dulbecco's modified Eagle's medium High Glucose, pyruvate-free (DMEM; Cellgro) supplemented with 10% fetal bovine serum (Invitrogen, Grand Island, NY, USA). Viability experiments were performed in serum-free media.

### Analysis of cell viability *in vitro*

Cell viability was assessed by Thiazolyl Blue Tetrazolium Bromide (MTT) assay as previously described [24]. Briefly 5, 000 cells/well were plated in 96-well plates and treated as indicated in the figure legends prior to assessing cell viability by MTT assay.

### Immunoblot analysis

Analysis of protein expression and/or phosphorylation was made by immunoblotting as previously described [25]. Briefly, 20 μg of protein were separated in 4–20% SDS-PAGE gels (Bio-Rad, Hercules, CA, USA) and transferred onto PVDF membranes. Membranes were blocked for 1 h at room temperature in 5% fat-free milk diluted in TBST (10 mM Tris-HCl, 100 mM NaCl, 0.5 M EDTA, 0.1% Tween 20). Primary antibodies diluted in 5% fat-free milk were incubated overnight at 4°C. After 3 washes, horseradish peroxidase-linked secondary antibodies (Sigma-Aldrich, St Louis, MO, USA) were incubated for 1 h at room temperature. Signal was detected using the ECL protein detection system (Pierce, Thermo Scientific, Rockford, IL, USA). PKCθ antibody was from Abcam (Cambridge, MA); antibodies for IRS1, IRS1-ser1101, HSF1, tubulin, Akt, and p-Akt were from Cell Signaling Technology (Danvers, MA, USA); HSP70 antibody was from Thermo Scientific.

### Glucose uptake assay

Glucose uptake was measured using a fluorescent non-metabolizable D-glucose analog 2-[*N*-(7-nitrobenz-2-oxa-1, 3-diazol-4-yl) amino]-2-deoxy-D-glucose (2-NBDG, Cayman Chemicals, Ann Arbor, MI) as previously described [26]. Briefly 3, 000 cells were plated in black-well 96-well plates and were transfected the day after with small interference RNA against PKCθ or PKCα (10 nM; Santa Cruz Biotechnology, Dallas, TX, USA) using Dharmafect 3 transfection reagent as recommended in the manufacturer instructions (Dharmacon, Thermo Scientific, Pittsburgh, PA, USA). Eight hours post-transfection, cells were treated as indicated (16 h). At the end of the treatment, cells were incubated for 45 minutes in KREB buffer containing 1g/L glucose in presence or absence of 20 μM 2-NBDG. Cells were then washed 2 times for 10 minutes with PBS to remove all residual extracellular 2-NBDG. The amount of 2-NBDG imported into the cells was measured by assessing fluorescence at 488 nm.

### HSF1 transcriptional activity reporter assay

HSF1 transcriptional activity reporter assay was performed as previously described [4].

### Statistics

Unless specified, all values are expressed as mean ± standard error. Values were compared using the Student-Newman-Keul's test. *P* < 0.05 was considered significant.
